# A HUG taxonomy of humans with potential in human–robot hugs

**DOI:** 10.1038/s41598-024-64825-8

**Published:** 2024-06-20

**Authors:** Zheng Yan, Zhipeng Wang, Ruochen Ren, Chengjin Wang, Shuo Jiang, Yanmin Zhou, Bin He

**Affiliations:** 1 Shanghai Research Institute for Intelligent Autonomous Systems, Tongji University, Shanghai, 201210 China; 2 State Key Laboratory of Intelligent Autonomous Systems, Shanghai, 201210 China; 3 Frontiers Science Center for Intelligent Autonomous Systems, Shanghai, 201210 China; 4 College of Electronics & Information Engineering, Tongji University, Shanghai, 201804 China

**Keywords:** Electrical and electronic engineering, Classification and taxonomy

## Abstract

Humans can easily perform various types of hugs in human contact and affection experience. With the prevalence of robots in social applications, they would be expected to possess the capability of hugs as humans do. However, it is still not an easy task for robots, considering the complex force and spatial constraints of robot hugs. In this work, we propose the HUG taxonomy, which distinguishes between different hugging patterns based on human demonstrations and prior knowledge. In this taxonomy, hugs are arranged according to (1) hugging tightness, (2) hugging style, and (3) bilateral coordination, resulting in 16 different hug types. We then further study the hug type preference of humans in different scenarios and roles. Furthermore, we propose a rule-based classification system to validate the potential of this taxonomy in human–robot hugs based on a humanoid robot with an E-skin of contact sensation. The HUG taxonomy could provide human hugging behavior information in advance, facilitating the action control of humanoid robots. We believe the results of our work can benefit future studies on human–robot hugging interactions.

## Introduction

The study of hugging behaviors in interactions is important across various disciplines. Hugs have significant correlations with human well-being and mental health^1^, offering benefits like anxiety relief^2^, and sociological support^3^. Essentially, hugs are pivotal and intentional behaviors^4^, manifesting in various scenarios^5,6^—social occasions, intimate relationships, emotional expressions, and movement functions. Unfortunately, ever fewer necessary hugs are happening due to increased workloads and the aging population, leading to an increasing number of people feeling lonely and depressed^7^. With the widespread robotic applications in social services^8^, providing high-quality hugging interactions by robots has proven to be a viable solution^9^. However, computational behavior research^10^ provides evidence that hugs are complex binary natural interactions. Spatial and force constraints of dual arms are influenced by various high-level social and biological factors. This poses a challenge for sensorimotor control systems of robots.

Current solutions for hugging robots either require remote operator control^11–13^ or only provide non-reciprocating, non-adaptive, or pre-defined embrace^14–16^. To ensure a positive user experience, hugging robots should not only be humanoid but also understand human body language and respond accordingly. Thus, exploring human hugging patterns is vital for enhancing human–robot hugs.

It is noteworthy that taxonomies are frequently employed to handle the complexity of robotic task execution^17–20^. The intricate interaction between humans and robots, coupled with the difficulty of dual-arm coordination, complicates the direct modeling of human–robot hugs. Nevertheless, the patterns of hugging behaviors can be divided into distinct subcategories^10^. However, the hugging behavior studies only focus on the influence of hugging variables, overlooking classifying the hugging patterns. To the best of our knowledge, few studies have attempted to categorize hugs with clear classification criteria and definitions. For these reasons, we incorporate prior knowledge of hugging behavior research across robotics, behavioral science, and psychology. Based on prior knowledge, we analyze demonstrations of human hugs in a motion capture system and summarize the key aspects to guide taxonomy construction. We aim to propose a taxonomy that supports the robotic learning of hugging patterns from human behaviors and the robotic execution of hugs.

To the best of our knowledge, for the first time, we propose a well-defined taxonomy of distinguishable hugs into 16 categories, for the simplification of hugging behaviors. Three key aspects (hugging tightness, hugging style, bilateral coordination) of human hugging behaviors are proposed to construct the HUG taxonomy explicitly. The comprehensiveness of the HUG taxonomy is further determined based on human demonstrations and the comparison of related works. To provide insights into human–robot hugs based on the HUG taxonomy, we analyze differences in hug preferences of different hugger roles and scenarios. The potential of the HUG taxonomy in human–robot hugs is validated, using the taxonomy-derived rule-based classification system on robots with E-skin. This work would provide useful information not only for control strategies that can be fluidly switched and implemented on humanoid robots but also for the anthropomorphism of robotic behaviors to optimize the experience of human–robot hugs.

All participants sign informed consent to participate in the research and to publish the identifying information/images in an online open-access publication. The ethical approval is provided by the Science and Technology Ethics Committee of Tongji University, following the Ministry of Science and Technology of the People's Republic of China. All experiments are performed in accordance with relevant guidelines and regulations.

## Methods

**Figure 1:**
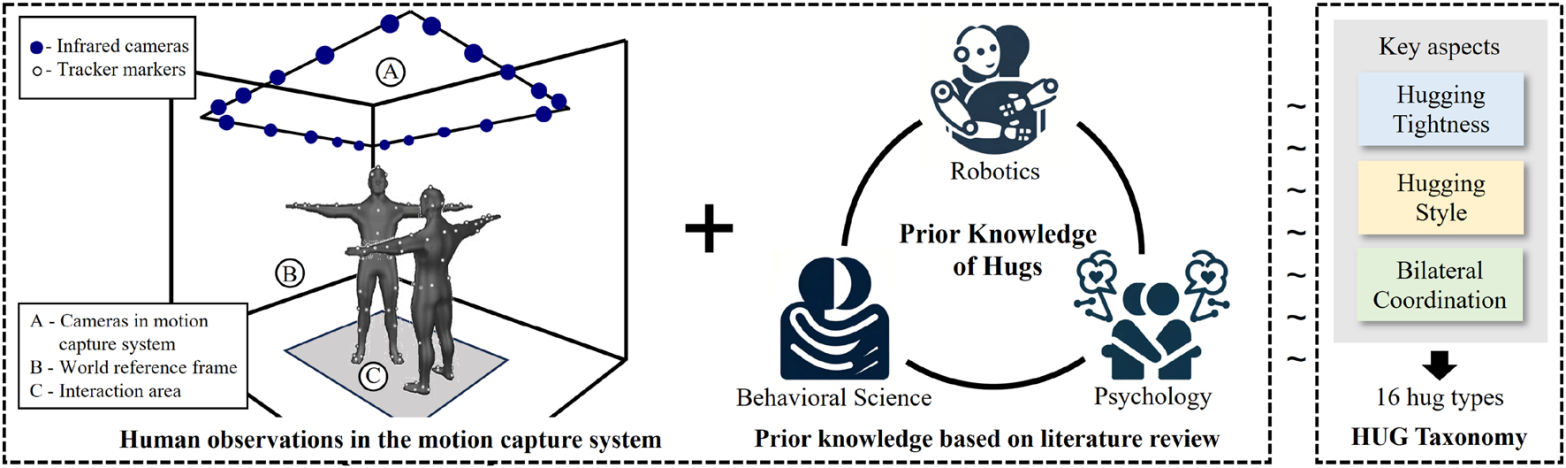
An overview of the proposal of the HUG taxonomy. HUG taxonomy (right) is proposed based on the human demonstration (left) and prior knowledge (middle).

Given that multiple participants are not always involved in hugs and challenges yet to be overcome in robotic operation, we do not consider multi-person hugs or single-arm hugs in this work. Hugs contain about 4 steps^21^, in which “the approach” and “the embrace” determine the hugging pattern. The approach process is influenced by the embrace posture that is of interest in this study: focusing on their upper body posture after the approach of individuals. We suppose that the hugging behavior patterns are related to embracing posture, while the duration of the hug does not affect it. Therefore, the temporal factors are not considered in this work. As shown in Fig. 1, we combine prior knowledge with human demonstrations to summarize the emergent physical properties of hugs to construct the taxonomy for hugging behaviors.

### Prior knowledge

Hugging behaviors have been studied in the light of anthropology, including many facets. For example, in kinesics, all bodily movements can be discriminated^22^. In proxemics, hugs were defined as “how we structure, use, and are affected by space in our interactions with others”^22^. Morris^23^ described hugs as, “… consists of a total embrace, bringing both arms around the friend’s body, with frontal trunk contact and head contact.”

Past research has delved into the taxonomy-oriented analysis of hugging behaviors. Floyd^24^ conducted a comprehensive review of massive videos of hugs, assessing factors such as positivity, egalitarianism, expectedness, and intimacy. The involved variables of the hugs in the videos encompass the arms-crossing style (criss-cross, neck-waist, engulfing) and gender. Floyd indicated that criss-cross hugs were perceived as more equal and intimate than neck-waist hugs, while gender nearly had no significant influence on the expectation of the hugs. Forsell^25^ proposed that factors affecting the popularity of hugs included body position, hand position, hugging duration, and pressure. Variations of these elements typically correlated with the intimacy and emotional expression of the participants. For these reasons, Dueren^26^ further explored the influence of these abovementioned elements on inducing positive feelings. The results revealed that long hugs were more appreciated than short ones, while criss-cross hugs dominated over neck-waist hugs with the latter more prevalent in intimacy. Additional research^27,28^ focused on hugging laterality, recording a general right-arm dominance in hugs from human observations.

The purpose of these human hugging studies was to analyze how humans evaluated different hugs and their prevalence. However, these studies only focused on the influence of hugging variables, overlooking posture-oriented hug types. Although a few studies^6^ have attempted to categorize hugs based on scenarios and meanings, clear classification criteria and definitions remained absent.

Despite the recent surge in researchers developing hugging robots, this field remains relatively nascent. Initially, researchers attempted to devise robotic systems focused on providing companionship without actively embracing the user. For instance, Stiehl^29^ designed a small robot (Huggable) resembling a teddy bear, which emulated stuffed animals to provide companionship and emotional expression, making it suitable for comforting children during hospital stays. DiSalvo^30^ introduced a pillow-shaped robot with the posture of a child reaching out his arms. This humanoid design has been proven to promote closer interpersonal interactions. These robots, although compact and safe, were limited in their capacity to provide real social touch and deep touch pressure therapy. Yoshimura^31^ and Kim^32^ demonstrated that the intensity and dual-arm position of hugs were crucial in human–robot emotional interaction.

Other researchers focus on the development of humanoid robots designed for providing hugs. Yamane has developed a realistic, real-time, and comfortable remote-controlled hugging robot^11,13^, though requiring the robot operator’s remote presence to hug users. Block and Kuchenbecker's team have been dedicated to developing the HuggieBot, a robot that could offer distinctly anthropomorphic and comfortable hugs^14^. They^15,33^ conducted a quantitative assessment of human evaluations of robot physical conditions (i.e., softness and temperature) and characteristics of hugging behaviors (i.e., duration and pressure). Further research^34,35^ emphasized humans' particular sensitivity to elements of hugs, notably the intra-hug gesture of the robot and the hugging tightness.

Human–robot hugs are highly dynamic and rapid interactions. The hugging robots that improve adaptive capabilities by practicing with users or performing predefined actions lack adaptive collaborations. Michael^36^ tried to address this challenge by introducing a human–robot interaction framework, containing a list of possible interactions of only three hug types. Considering the different dynamics between the initiator and receiver of a hug, and the corresponding nature of a hug to specific situations, there are many variations and types of hugs. Therefore, the insight into the differences between hugs can not only enhance the anthropomorphic and adaptiveness of robot hugging behavior but also be crucial for enhancing human understanding and trust in hugging robots.

### Human demonstrations

**Figure 2:**
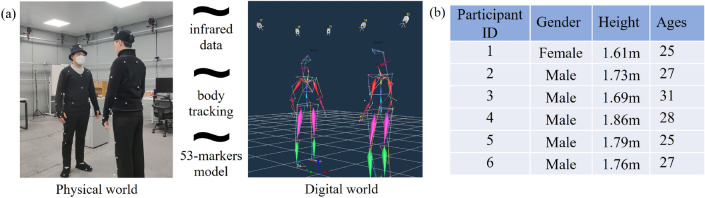
Motion capture experiments of the human demonstration. (**a**) Motion capture system. (**b**) Participant information.

We have achieved prior knowledge that is the focus of research on human hugging behaviors as well as hugging robots. Based on prior knowledge, we conduct human demonstration experiments and collect motion capture data to further analyze the emergent physical attributes of hugging behaviors. As shown in Fig. 2a, we utilize the motion capture system from NOKOV to collect data. It consists of 20 infrared cameras with 60fps, arranged in a square on the ceiling of a *6.5m×6.5m×2.5m* quiet room, with five cameras on each side. Combined with the 53-marker human body detection and modeling algorithms, human demonstrations can be transformed into digital models, making physical properties more apparent. To ensure the quality of human demonstration data, the participant group mainly consists of young adults. We record the demographic information (heights, genders, and ages) of the participants in the experiment, as shown in Fig. 2b.

Initially, two participants, wearing motion capture suits with 53 markers, are requested to freely hug each other. If they have independently completed all the hugs they could conceive, we will provide scenarios as guiding prompts based on prior knowledge. Scenarios include social occasions (e.g., meeting among friends^6^), intimate relationships (e.g., greeting or farewell among couples or family^6^), emotional expressions (e.g., expressing intense anxiety or happiness), and movement functions (e.g., leading to movement, like rotation or panning.). We also consider roles (initiators and receivers) in the experiments. Before each hug, participants should discuss the roles they are assuming, as well as their behaviors which might involve planned hugs or even surprise hugs. This ensures the authenticity of hugging behaviors. The initiator decides when to start the hugging process, and then the receiver responds accordingly based on prior information, instinct, and habit.

Each participant engages in a hug demonstration experiment with every other participant, resulting in 15 unique pairings. Within each pairing, each participant plays two hugger roles during the experiments. This is recorded in real-time by the motion capture system, resulting in 379 samples. We record hugs with no complete hug process as failed ones, thus not included in the dataset. The distribution of all participants' samples can be seen in Supplementary file S5.

The occurrence of hugging behaviors is influenced by various factors, including but not limited to motivation (which is often driven by scenarios and roles) and participants' states (such as personality, emotions, gender, height, and cultural background). In the proposal of taxonomy, we consider these factors that influence hug type choices through prior knowledge and human observation. However, we focus on the emergent physical properties of the hugging behavior, which we use to construct hug types in the taxonomy. Regardless of the motivation and the participants’ states, the completion of hugging behaviors would not be affected. Therefore, based on the key aspects of human hugs, we determine the emergent properties of each hug type to construct the proposed taxonomy. However, we would then analyze the relationship between the hug types and the motivation, regardless of the participants' states.

## Results and discussion

### HUG taxonomy

#### Key aspects

Based on prior knowledge and human demonstrations, we discuss and elaborate on the key aspects that must be considered in the taxonomy and their properties. These key aspects include hugging tightness, hugging style, bilateral coordination, and dominant side.

**Hugging tightness:** In the field of robotics, it is common and reasonable to consider hugs as large-scale two-finger grasps where the object to be grasped is human^14^. Grasps are often classified based on precision or power requirements^18^. Combining with Forsell’s work^25^, we propose a similar criterion, delineating hugs with two categories: *body hug* and *air hug*. In the *body hug*, the arms of one hugger are tightly clasped around the other's body, making full contact. It means that their actions are interdependent and mutually influenced. The *air hug* is characterized by body detachment between huggers, with flexible connection and little contact. Here, individuals can easily disengage or adjust their positions. This criterion is related to hugging squeeze as defined by pressure constraints, which can be represented as the physical contact between participants. In our taxonomy, *body hugs* and *air hugs* appear in roughly equal proportions. While hugging tightness allows for finer differentiation of hug types, the foundational distinctions remain consistent.

**Hugging style:** Considering the asymmetry in heights between participants, binary hugs can be typically categorized into two main types based on hugging style: criss-cross hug and neck-waist hug^26^. Based on the specific execution of the individual, the neck-waist hug can be further subdivided into waist-loop hug and neck-loop hug. The criterion focuses on the spatial alignment constrained by the comfort of huggers, which is largely presented in the differences in their upper arms positions, regardless of the forearms.

- *Neck-loop hug* sees one hugger wrap his arms around the neck of the other, with both upper arms raised.
- *Waist-loop hug* sees one hugger wrap his arms around the other's waist, evident by both upper arms downwards.
- *Criss-cross hug* is enacted by having one hugger put one arm up and the other arm down, noticeable by the differing upper arm paths.

**Figure 3:**
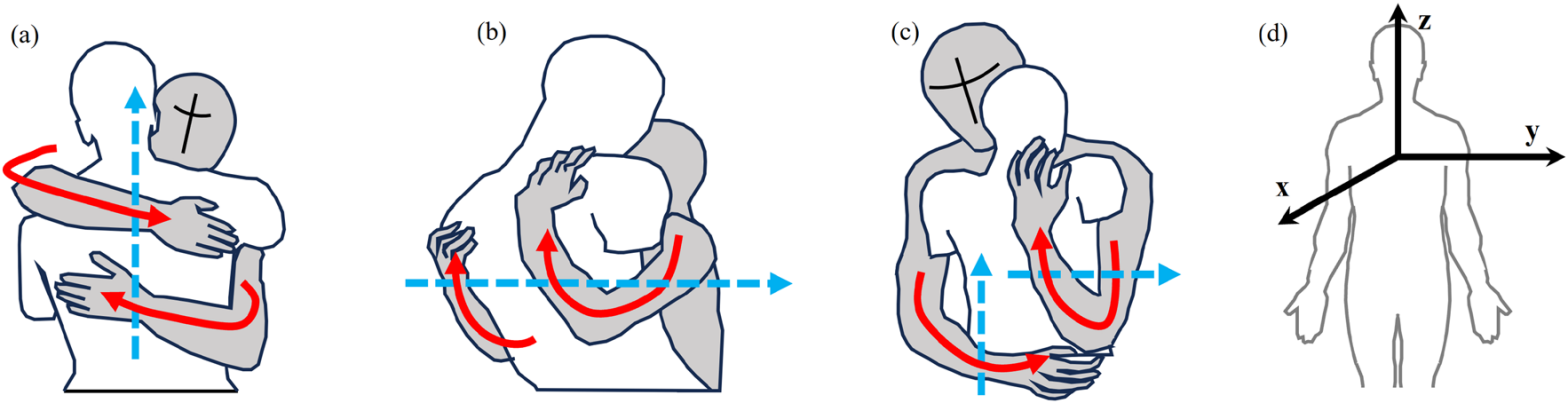
Bilateral Coordination examples of hugs. The solid red lines represent the outline of arms, and the dashed blue lines represent the axis around which dual arms surround. (**a**) is ‘horizontal parallel hug’, (**b**) is ‘vertical parallel hug’, (**c**) is ‘perpendicular hug’, and (**d**) is a human coordinate system.

**Bilateral coordination:** Observed in hugging behaviors, while the postures of dual arms are distinctly varied and complex, there is a dependency on potential spatial and force constraints. In the human coordinate frame, to hug comfortably, there are three primary directions around which one hugger can apply forces on the other’s body. It means that one hugger's arms and chest form a loop around the other, and the axis of the loop can be typically two directions: one usually occurs along the direction of the body, represented as the z-axis in Fig. 3d; one occurs along a direction generally transverse to the body, represented as the y-axis in Fig. 3d. Based on our surveys^18^ and observations, we have identified three spatial and force application patterns of dual arm:

- *Horizontal parallel hug* (see Fig. 3a): one hugger wraps his arms around the other along the z-axis, with forearms placed parallel on the other's back.
- *Vertical parallel hug* (see Fig. 3b): one hugger wraps his arms around the other along the y-axis, with forearms parallel across the other's back.
- *Perpendicular hug* (see Fig. 3c): one hugger wraps his arms around the other along the y-axis and z-axis respectively, with the forearms crossing over the other's back.

**Dominant side:** Research^27^ indicates an obvious asymmetry in hugging behaviors with a population-level lateral preference, where adults predominantly choose their right side for hugging. This asymmetry is linked to a combination of motor preferences and emotional distinctions. When emotions are under control, motor preference might explain the variation between individual hugs. However, hugs typically occur in emotional contexts, which can subtly affect hugging asymmetries. This can be explained under the assumption that neural networks involved in emotional and motor processing are intertwined. Since the right hemisphere exhibits motor control of the contralateral body half, a general activation of right-hemispheric networks through the affective state provides the most persuasive explanation for these results. However, lateral biases in hugs usually only lead to the symmetry changes of the dual-arm posture. Thus, we do not distinguish hugging side bias.

#### Taxonomy matrix

**Figure 4:**
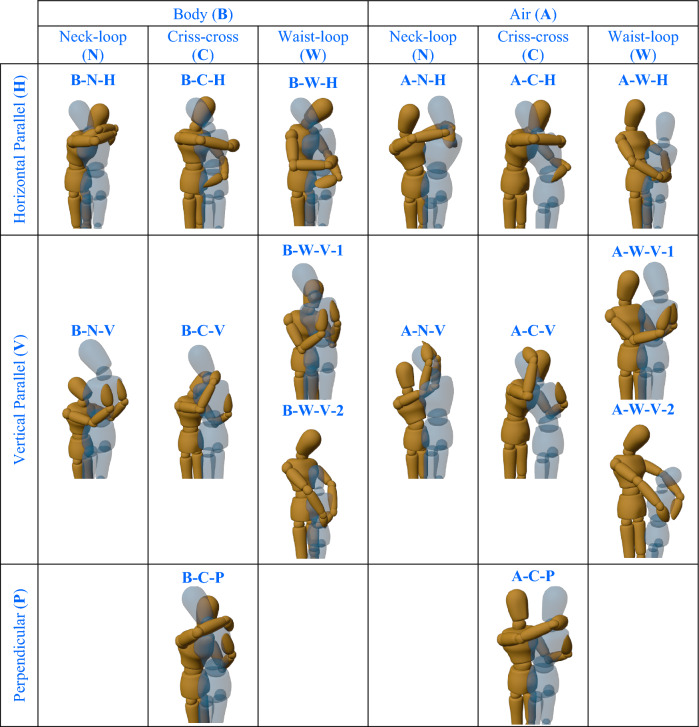
HUG taxonomy that all hug types are arranged in the matrix. The pose of brown puppets (rather than blue puppets) is the concerned hug type. To clearly show the details of the hug types, the blue puppet is transparent with no arms. These photos are from the same view of the 3D model, which is motion capture data modeling results based on the Blender.

Based on the deliberation and determination of the properties in key aspects of human hugging behaviors, we propose a taxonomy that classifies hug types in a matrix arrangement from one hugger perspective (see Fig. 4). The columns are firstly arranged according to hugging tightness (*body*, *air*). The next finer distinction within each column depends on the hugging style (*criss-cross*, *neck-loop*, or *waist-loop*). Hugs are further distinguished by spatial and force application patterns of the dual arm (*horizontal parallel*, *vertical parallel*, or *perpendicular*).

The HUG taxonomy describes the posture of the individual hugger, while each of the hugging participants performs a hug type to constitute a hugging behavior. In addition to face-to-face hugs, hugs that are distinctly unique due to the specific positional relationship of the huggers can also be formed by combining the types in the HUG taxonomy, as follows:

- *Reverse hug*: the hug initiator approaches and wraps his arms around the hug receiver from behind, allowing for all body hug types. The receiver places his hands over the arms of the initiator, maybe leaning back and resting his head against the initiator.
- *Side-twist hug*: two huggers, constrained to be positioned side-by-side, twist towards each other to do a face-to-face hug. In this case, the hug types chosen by huggers tend to be the same as face-to-face hugs.

#### Taxonomy completeness

It is important to determine the completeness of the HUG taxonomy^18^. We answer this question via the human demonstration dataset and the comparison with research on hugging behavior.

**Figure 5:**
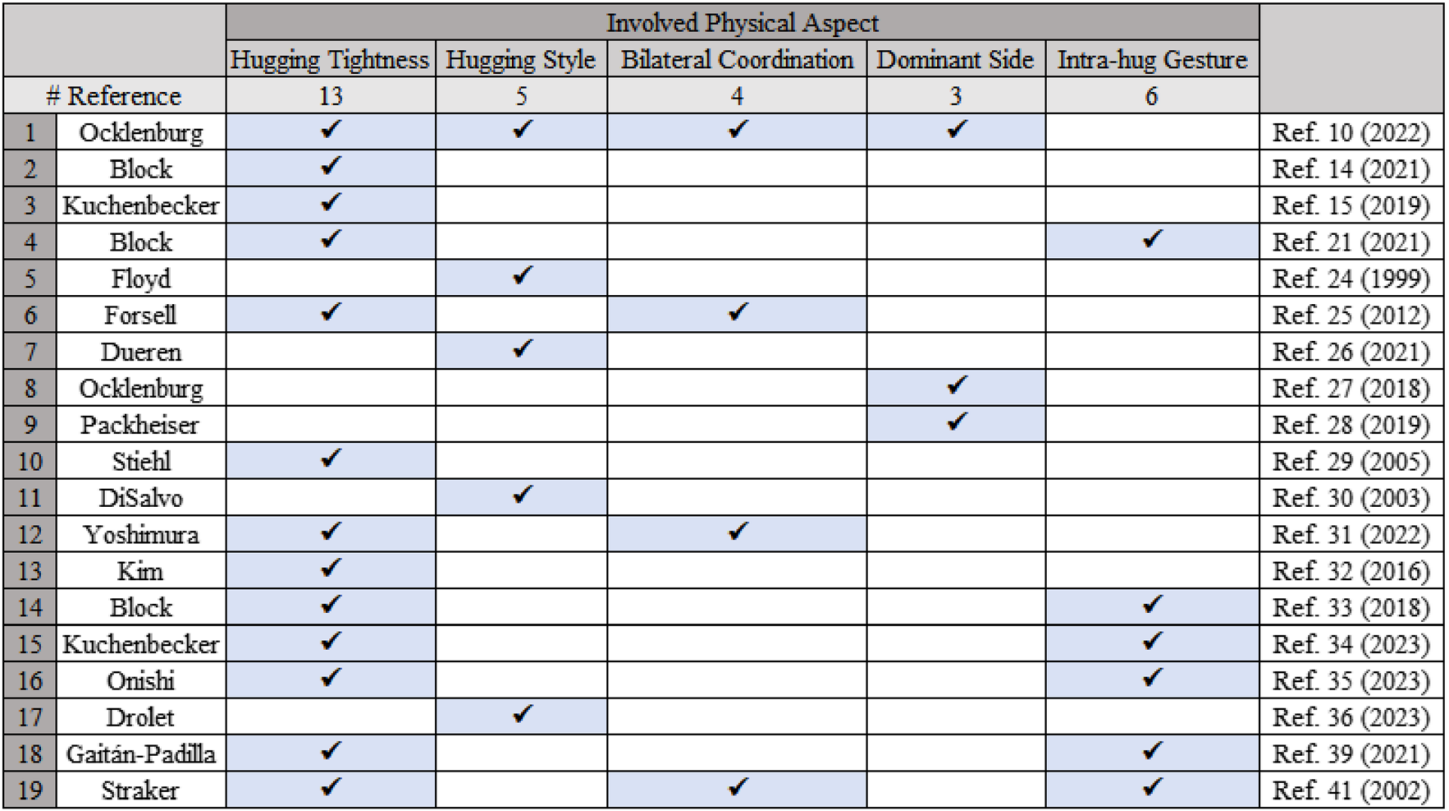
Comparison of the physical aspects involved in the 18 publications. In particular, the dominant side and intra-hug gestures are not involved in the HUG taxonomy, but we explicitly discuss these two aspects in our work.

To the best of our knowledge, the HUG taxonomy is the first well-defined taxonomy of hugs. Therefore, we compare the HUG taxonomy with the hugging behavior research (see ^10,14,15,21,24–38^), covering the domain of humans and robots. Most reviews of the human hugging behavior research only suggest taxonomic ideas without conducting any further classification. In the hugging robot studies, we summarize the factors that they consider in the hugging robot design. To facilitate comparison, all studies are registered in a comparison table, where rows store all physical aspects considered by the authors. The aspects are considered equivalent if the involved properties defined are similar. As shown in Fig. 5, the proposal of the HUG taxonomy considers all the involved physical aspects of hugs in these studies. At the same time, we describe the definition and classification of key aspects in the HUG taxonomy.

Subsequently, we record the frequencies of hug types during the human demonstration. The distribution of different types of hugs within the human demonstration dataset is shown in Table 1. It is noted that each human demonstration hug sample contains two hugs, i.e., 758 hugs in the human demonstration dataset. The results show that our proposed taxonomy can distinguish all hugs present in human demonstrations, and all hug types are used. We believe these results to some extent indicate the comprehensiveness of our taxonomy.

**Table 1:** The distribution of different types of hugs within the human demonstration dataset.

Hug type	A-C-H	A-C-P	A-N–H	A-N-V	A-W–H	A-W-V-1	A-W-V-2	A-C-V
Count	102	51	31	10	101	57	12	56
Percent	13.46%	6.73%	4.09%	1.32%	13.32%	7.52%	1.58%	7.39%
Hug type	B-C-H	B-C-P	B-N–H	B-N-V	B-W–H	B-W-V-1	B-W-V-2	B-C-V
Count	88	46	19	4	102	26	9	44
Percent	11.61%	6.07%	2.51%	0.53%	13.46%	3.43%	1.19%	5.80%

Furthermore, it is noted that categories in the HUG taxonomy could encompass several hugs. For instance, in B-C-H hugs, two arms may constitute a partially closed loop, fully closed loop, or even in contact. However, this level of hug classification can already guide robot control. For example, by setting appropriate joint thresholds, the robot can safely embrace users^14^. Therefore, it is reasonable to simplify these similar hugs into one hug type in the HUG taxonomy. Adding subtle variations (maybe intra-hug gestures) offers the possibility for further hug-type distinction for huggers of different states^34^.

In the HUG taxonomy matrix, there are 4 cells, which are not populated with any hug. One reason is that these hugs can be conceivable but barely observed in human demonstrations, e.g., neck-loop (waist-loop) hugs with the arms perpendicular. Another reason is that hugs that are potentially inaccessible are not involved. These hugs are not feasible in human habits, e.g., when the upper arm of the hugger is raised, they do not usually keep the forearm down.

Additionally, see Supplementary file S1, we question eight subjects to show them different subsets of HUG taxonomy in the matrix, separately. Then we check the correlation of the agreement with all the given subsets and see if the subjects would correctly infer the missing hug types that our taxonomy assumes. The results show that all subjects consider the given subsets to be incomplete. For a subset lacking properties, subjects could point out the missing properties in the key aspects. However, it is difficult for them to infer all the missing hug types if a subset includes all properties. We believe this result is due to the rarity of specific hug types, as shown in Table 1, even though they are reasonably present. Taken together, we suppose that the HUG taxonomy encompasses an exhaustive range of key aspects and hug types.

### Statistical analysis of HUG taxonomy

The HUG taxonomy is derived from observing human hugs and reviewing the literature, the involved hug types are the consequence of a combination of three aspects (hugging tightness, hugging style, and bilateral coordination). Generally, certain aspects take precedence over others, yet there is no definitive consensus on which aspects are paramount in categorizing hug types. As we mentioned, the hug type choice is influenced by motivation and participants' states. Here, we only analyze how hug type correlates with motivation (different scenarios and roles) to provide insights into human–robot hugs.

We obtained 16 hug types in the HUG taxonomy and their corresponding depicts and 3D models, (see Supplementary file S2). We then conducted a questionnaire study, considering that surveys always include "rude" participants, all the participants were recruited and checked volunteers. None of the participants knew about the HUG taxonomy. When they answered the questionnaire (see Supplementary file S3), the authors recorded the demographic information of participants and accompanied them throughout the process. A total of 36 individuals participated in the survey: 75% were male and 25% were female. Participants ranged in age from 19 to 52 years old (mean = 28, standard deviation = 7.11). Most participants indicated that they had an interest in robots (97.22%) and had experience in physical interaction with robots (77.88%).

Participants first viewed depicts and 3D models of 16 distinct hug types as many times as they liked. They were then posed with the first question, "Do you believe these 16 categories encompass all the hugs you would make?" If their response was negative, a space would be provided to describe any missing hug type. Subsequently, they rated questions on a 5-point Likert scale to indicate their preferences of hug types as initiators in any given scenario. Finally, they should indicate which hug types they would prefer for a response when facing each of the 16 hug types from initiators.

Results of the first question indicate that most participants (95%) acknowledge the completeness of the HUG taxonomy. Only 2 individuals suggest the lack of the hug type: princess hug (i.e., the initiator holds the receiver horizontally across his chest). However, considering its dual-arm posture, the princess hug is in line with the hug type A-W-V-1 or B-W-V-1.

**Figure 6:**
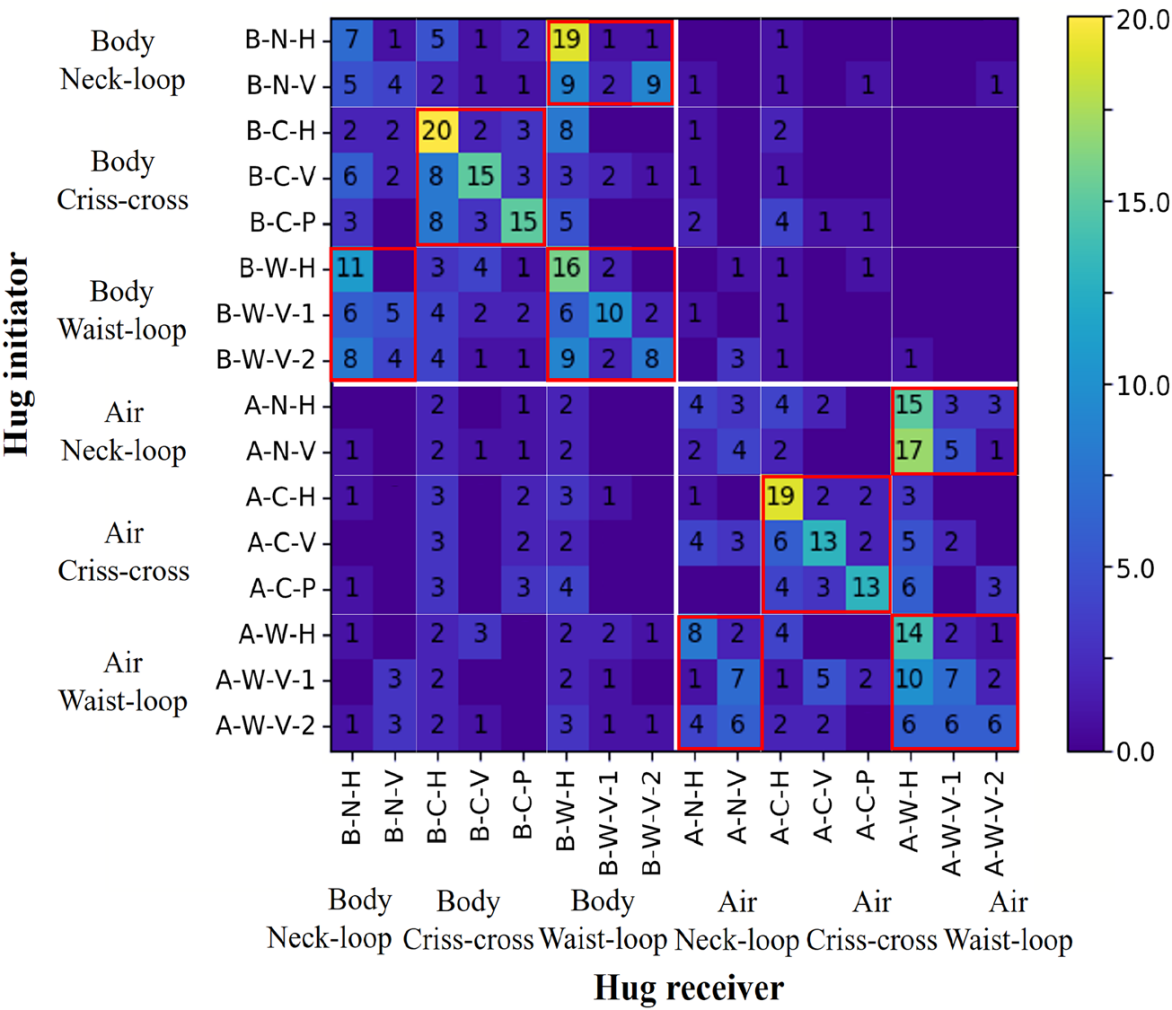
Comparison of hug types between the hug initiator and the hug receiver. The white boxes distinguish body hugs and air hugs between the initiator and the receiver. The red boxes indicate the most common combination of hug types chosen by the initiator and the receiver during all scenarios.

In the survey, we first focus on the hug-type preference of scenarios among participants, when as the initiators. Then, participants, when as the receivers, could choose several hug types in response to the initiators. This scenario information is involved throughout the questionnaire process. Figure 6 shows all the combinations of hugs of the initiator and the receiver. The most frequent occurrences are found on the diagonal of this map, which displays a high similarity in the hugs. However, the top-right semi-plane is more populated than the bottom-left semi-plane, showing the tendency of receivers to use more *body hugs* than initiators. *Neck-loop hugs* and *waist-loop hugs* are usually coupled, while *waist-loop hugs* could also match the same hugging style.

**Figure 7:**
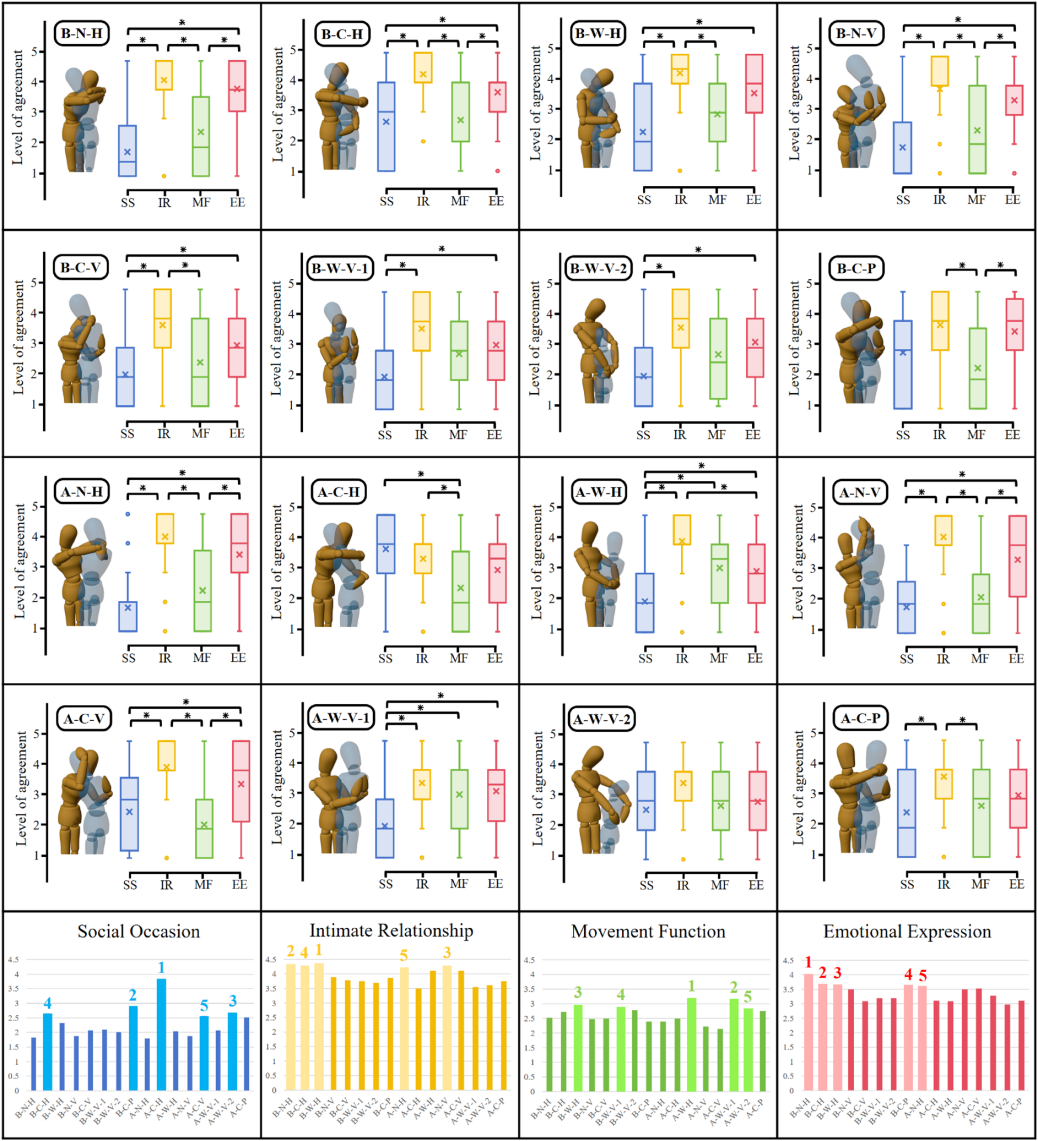
Distribution of hug type choice of the hug initiator relative to scenarios. SO represents social occasions, IR represents intimate relationships, MF represents movement functions, and EE represents emotional expressions. The significant comparisons (P < 0.01) are reported with (*). All the statistical analysis results are provided in the Supplementary file S4.

Figure 7 presents the answers to the 5 Likert-style questions. For all statistical analyses, we utilize the repeated measures ANOVA Friedman test. Then, we apply the Bonferroni alpha correction with α = 0.01 to account for multiple comparisons, with Cohen’s d value to report effect size. The results show that hugging tightness is predominant in social occasions and intimate relationships. The hugging style is important in all scenarios. The bilateral coordination dominates in all scenarios except for movement functions. The 4 graphs in the last row of Fig. 7 illustrate the results of the statistical analysis for all hug types in the four scenarios. For each scenario, there are statistically significant differences in different hug types. The scores for each hug type are ordered based on the mean value, with the top five hug types marked. Hug A-C-H, hug B-W–H, hug A-W–H, and hug B-N–H appear the most in social occasions, intimate relationships, movement functions, and emotional expressions.

### Robotic potential of HUG taxonomy

**Figure 8:**
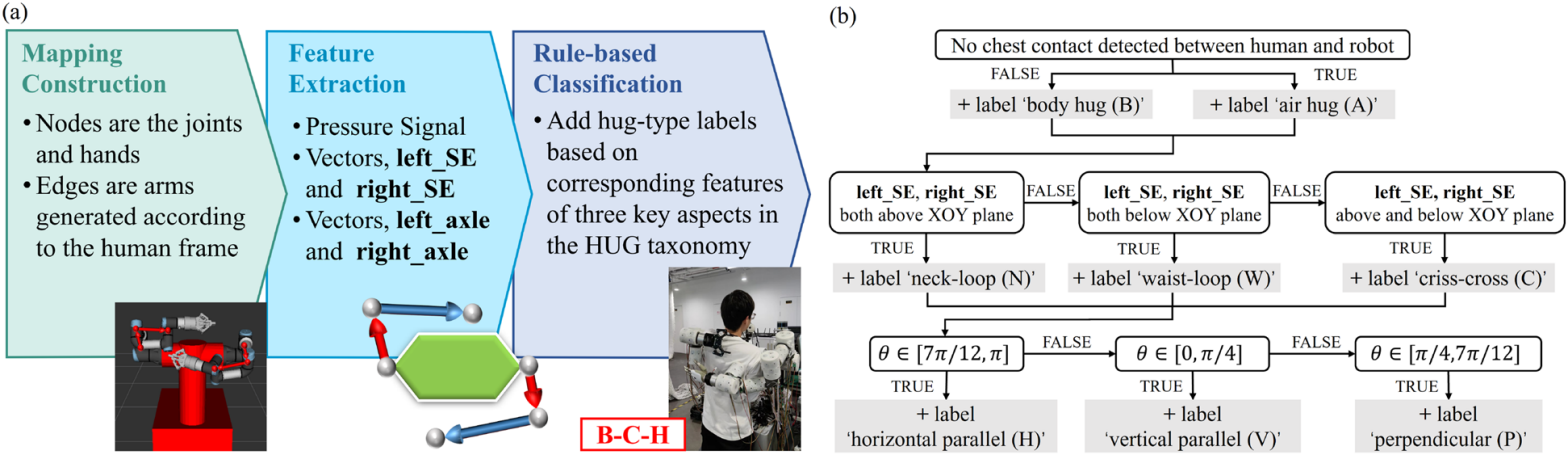
Rule-based classification system. (**a**)The figure shows an example of the mapping with E-skin signal (shown as a hexagon), as well as the detected hugging category as B-C-H hug. (**b**) Decision tree for distinguishing hug types.

To demonstrate that the proposed taxonomy derived from human behavior can also be applied to robots^17^, we propose a method based on the taxonomy for distinguishing robotic hugging patterns. This would provide data-driven validation for the taxonomy implemented in robots. To achieve this goal, we introduce a pipeline that integrates data processing, feature extraction, and classification of robot postures into different hug types (see Fig. 8). The 3D poses and configuration of robotic arms, and E-skin signals on the robot chest are used as input. In the first step, we construct a mapping from UR3s to human-arms-like structures. In the second step, the features are extracted based on the mapping, which would be used in the third step of a rule-based classification to identify hug types.

#### Rule-based classification system

During the robot executions, it is important to determine the degree of body contact and the spatial relationship between the arms. For the robot, we map it to the human’s framework (as shown in Fig. 8a), leveraging joint angles and rod lengths. The mapping is constructed by first evaluating the structure of the robotic arm, then assigning joints to different nodes and clustering close-situated joints under the same node. Here, mapped from UR3s to human arms-like structures, nodes symbolize virtual joints and links between them represent virtual arms. Each node has a unique ID: **S** for shoulder, **E** for elbow, and **W** for wrist. Each link has a unique ID: **SE** for the vector from the **S** node to the **E** node, and **EW** for the vector from the **E** node to the **W** node. This method is universal and works for different robotic arms to construct such mappings.

To determine the hugging categories, we apply a rule-based classification derived from the HUG taxonomy by which the decision is made via predefined and interpretable if-else rules. In other words, it is an implementation of the HUG taxonomy on a robot.

The decision tree and rules are shown in Fig. 8b. In our method, the hug type is determined according to the mapping topology and E-skin signals, with the corresponding feature of key aspects from the HUG taxonomy: Hugging tightness—the pressure signal from E-skin; Hugging style—vectors (**left_SE**, **right_SE**) of two arms; Bilateral coordination—vector cross products (**left_axle**, **right_axle**) of **SE** and **EW** of two arms. The specific implementation process is as follows:

- We start from the root node of the decision tree and check whether the robot’s chest is in contact, i.e., whether there is a pressure signal from the E-skin. If so, it belongs to *body hugs*, otherwise *air hugs*.
- Next, **left_SE** and **right_SE** are compared and used for decisions on the next level of the tree. If both vectors are below the XOY plane, the ‘*waist-loop*’ label is added; if both vectors are above the XOY plane, the ‘neck-loop’ label is added; otherwise ‘criss-cross’ label is added.
- Finally, the angle θ between left_axle and right_axle is calculated for decision-making in the last layer of the tree. While θ∈[7π/12, π] label the hug ‘horizontal parallel’; θ∈[7π/12, π] label the hug ‘vertical parallel’; and θ∈[π/4, 7π/12] label the hug ‘perpendicular’.

#### Experiment result

We conduct the human–robot hug experiment using a humanoid robot^39^ with two Universal Robot 3 (UR3) as arms. The robot is covered with electronic skin (TacSuit)^40^, developed independently by our team, to provide multi-modal tactile sensation. TacSuit is composed of hexagonal units with a 15 mm side length, providing digital output in force, proximity, acceleration, and temperature. We also achieve an easy tactile display based on LEDs. The TacSuit unit displays blue when working normally without stimulation, purple when the human approaching is detected, and green when pressure is detected.

People who participated in the human demonstration experiment were requested to participate in the robotic experiment, for a total of six participants. They were asked to hug the humanoid robot in two situations: (1) Humans initiatively embraced the robot and chose their hug type freely. The robot responded after the approach of humans. (2) The robot actively performed the hug type instructed by the humans to invite them to approach and embrace.

It is worth noting that the human–robot hug experiment in this section is only for verifying the potential of HUG taxonomy in human–robot interaction. Therefore, the experiments contain the following steps: (1) The human operator of the robot determines the hug types of the robot by interviewing participants. (2) The human operator enables the robot to complete specific hugging actions through kinesthetic teaching. (3) Human–robot hug is implemented through the human operator and participant cooperation practice. The detailed experimental process is shown in Supplementary file S6.

Before each experiment, the robot started with its arms hanging beside the body. Then, we recorded each hug by recording the robot's hold posture (joint angles, rod lengths, and E-skin signals), resulting in 125 samples. While recording the data, we manually labeled them according to the HUG taxonomy. The labels represented the categories of robot hugs.

From the manually labeled reference data, *air hugs* (52%) are more frequent than *body hugs* (48%), which might be affected by the rigid shape and appearance of our humanoid robot. *Criss-cross hugs* have the highest frequency (41.9%), followed by *waist-loop hugs* (32.3%), and *neck-loop hugs* (25.8%). *Horizontal parallel hugs* occur the most frequently (67.7%), followed by *vertical parallel hugs* (18.3%), and *perpendicular hugs* (14%). The rarity or even absence of hug types C-V and N-V may be due to the general lack of preparation of participants for head contact by robots. Table 2 shows the estimated accuracy and frequency for each hug type when the robot is the receiver, with an average accuracy of 95.4%. Table 3 shows the estimated accuracy and frequency for each hug type when the robot is the initiator, with an average accuracy of 96.6%.

**Table 2:** The accuracy and frequency of situation 1.

Hug type	A-C-H	B-C-H	A-C-P	B-C-P	A-N–H	B-N–H	A-N-V	B-N-V	A-W–H	B-W–H	A-W-V	B-W-V
Accuracy	87.5%	100%	100%	100%	100%	100%	100%	50%	100%	100%	100%	75%
Frequency	12.1%	15.2%	7.1%	7.6%	12.1%	7.6%	3.1%	3.1%	13.6%	7.6%	4.6%	6.3%

**Table 3:** The accuracy and frequency of situation 2.

Hug type	A-C-H	B-C-H	A-C-P	B-C-P	A-N–H	B-N–H	A-N-V	B-N-V	A-W–H	B-W–H	A-W-V	B-W-V
Accuracy	100%	100%	100%	100%	100%	100%	100%	100%	100%	66.7%	100%	100%
Frequency	12.1%	15.5%	6.9%	6.9%	13.8%	6.9%	1.7%	3.4%	8.6%	10.3%	8.6%	5.3%

The distinguish failures may come from two reasons: (1) Manually labeled data is used as ground truth data, this labeling is far from perfect as UR3s are not as flexible as human arms; (2) The division of in rule-based classification involves hyperparameters, angle θ which might be various with a different robotic arm. By using a more flexible robotic arm and adjusting hyperparameters, a better implementation of HUG taxonomy can be transplanted to robots.

However, the results have demonstrated that the HUG taxonomy can distinguish between different hug types both on robots and humans. It is reasonable to assume that it has the potential to be applied to robots (1) to predict hug types of participants, (2) to execute flexible human-like hugging behavior patterns, and (3) to make them more trustworthy to be hugged.

## Conclusions

We propose an explicit HUG taxonomy based on extracting key aspects from prior knowledge and human hugging observation, leading to 16 unique hug types. Based on human demonstration samples and the comparison of related works, we preliminarily determine the completeness of the HUG taxonomy. Then we analyze the influence of roles and scenarios in the hug type choice. Finally, we design a rule-based classification system derived from the HUG taxonomy to verify its potential on a humanoid robot with tactile E-skin. The HUG taxonomy could simplify hugging intent recognition and complex operations in human–robot hugs. Furthermore, it not only provides insights into human hugging behaviors but also informs robotic control strategies about how human habits migrate to robots.

Therefore, applying the HUG taxonomy to adaptive hugs, which mainly includes three aspects, will be the future of our work. (1) In “the approach” step, hugs can be initiated anytime with a starting point that is not predetermined. In this process, adaptive coordination is essential and requires the robot to predict the partner’s hug types and upcoming actions^36^. To address this issue, we would study embodied perception based on head vision and whole-body tactile sensation. (2) In “the embrace” step, we would attempt to guide the encoding that is essential for robots to perform hugs based on the HUG taxonomy. Such representation could allow the selection of appropriate controllers for different hugs, as well as switching and smooth transitions between different control strategies. This will account for an interaction that is a combination of multiple actions. (3) In the “release” step, we would apply touch or pressure signals in the hug release methods^37^.

It is worth noting that migrating the HUG taxonomy from human–human hugs to human–robot hugs can enhance human trust in hugging robots by explaining their behavior. We should also consider safety aspects, as well as the comfort, appearance^41^, and softness of hugging robots. We believe the HUG taxonomy will be instrumental in advancing the field of intimate human–robot hugging interaction.

### Supplementary Information

Supplementary Information 1.

Supplementary Information 2.

Supplementary Information 3.

Supplementary Information 4.

Supplementary Information 5.

Supplementary Information 6.

## Data Availability

All motion capture data are available upon request from the corresponding author.
