# Supplementary material for: A HUG taxonomy of humans with potential in human–robot hugs
Source: Sci Rep. 2024 Jun 20;14:14212. doi: 10.1038/s41598-024-64825-8 (PMC11190144; doi:10.1038/s41598-024-64825-8)
Supplement: Supplementary file 1 — Supplementary Information 1. [file 41598_2024_64825_MOESM1_ESM.pdf]

关于分类法完备性的调查

Survey on the completeness of HUG taxonomy

(We set up eight different subsets of HUG taxonomy (corresponding to eight questions) to ask subjects, and each subject answers only one question.)

Q1. 您认为这 8 个类别包含了您会完成的所有拥抱吗？（Do you believe these 8 categories encompass all the hugs you would provide?） [多选题] *

如下展示了拥抱类型的细节和描述（棕色木偶的姿势）
(The following figure shows the details and description of the hug types (the pose of the brown puppet).)

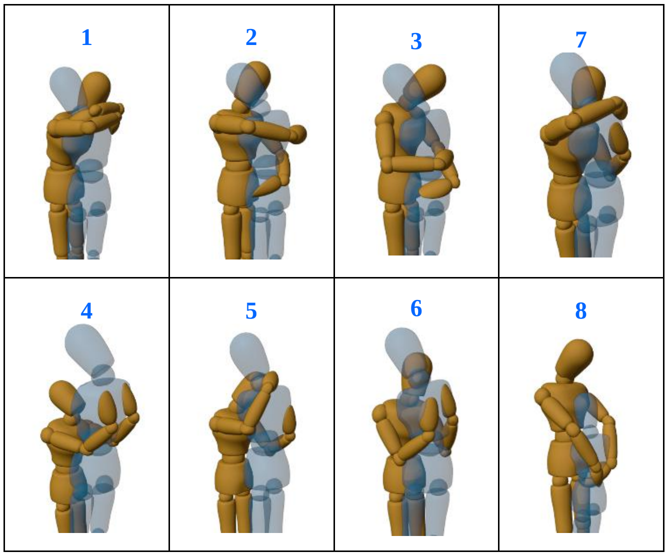

□如果是，选择此项即可（If so, select this option）

□如果否，请补充说明缺失的拥抱类型（If not, please describe the lack hug type）

____

Q2. 您认为这 14 个类别包含了您会完成的所有拥抱吗？（Do you believe these 14 categories encompass all the hugs you would provide?） [多选题] *

如下展示了拥抱类型的细节和描述（棕色木偶的姿势）
(The following figure shows the details and description of the hug types (the pose of the brown puppet).)

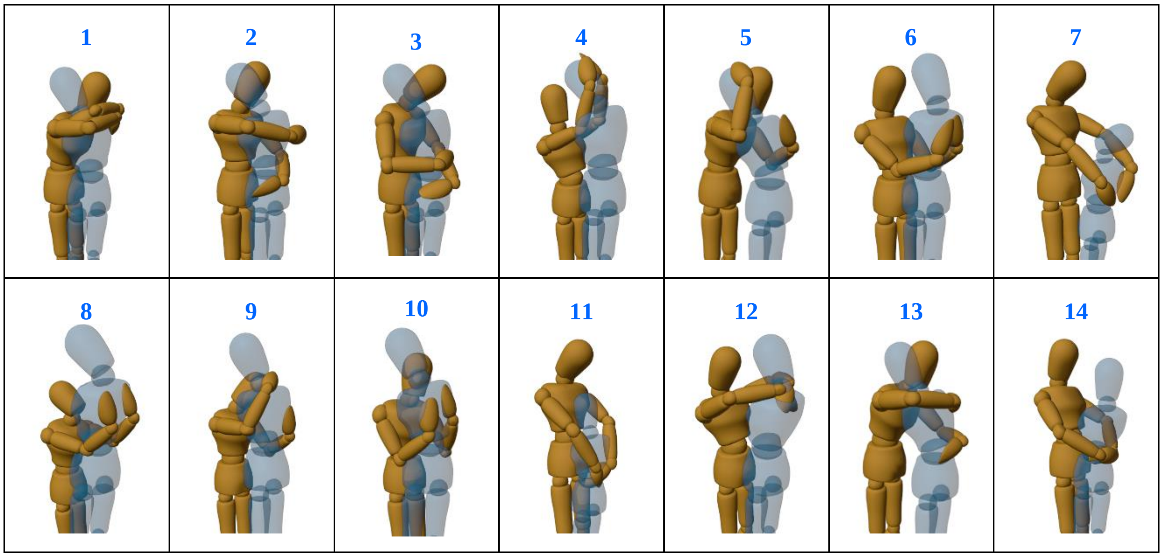

□如果是，选择此项即可（If so, select this option）

□如果否，请补充说明缺失的拥抱类型（If not, please describe the lack hug type）

Q3. 您认为这 12 个类别包含了您会完成的所有拥抱吗？（Do you believe these 12 categories encompass all the hugs you would provide?） [多选题] *

如下展示了拥抱类型的细节和描述（棕色木偶的姿势）
(The following figure shows the details and description of the hug types (the pose of the brown puppet).)

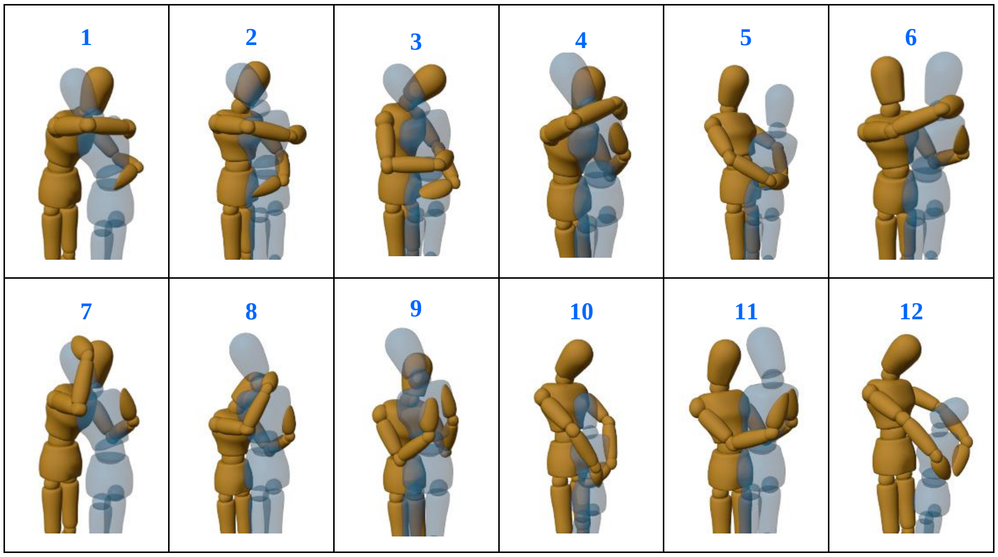

□如果是，选择此项即可（If so, select this option）

□如果否，请补充说明缺失的拥抱类型（If not, please describe the lack hug type）

____

Q4. 您认为这 10 个类别包含了您会完成的所有拥抱吗？（Do you believe these 10 categories encompass all the hugs you would provide?） [多选题] *

如下展示了拥抱类型的细节和描述（棕色木偶的姿势）
(The following figure shows the details and description of the hug types (the pose of the brown puppet).)

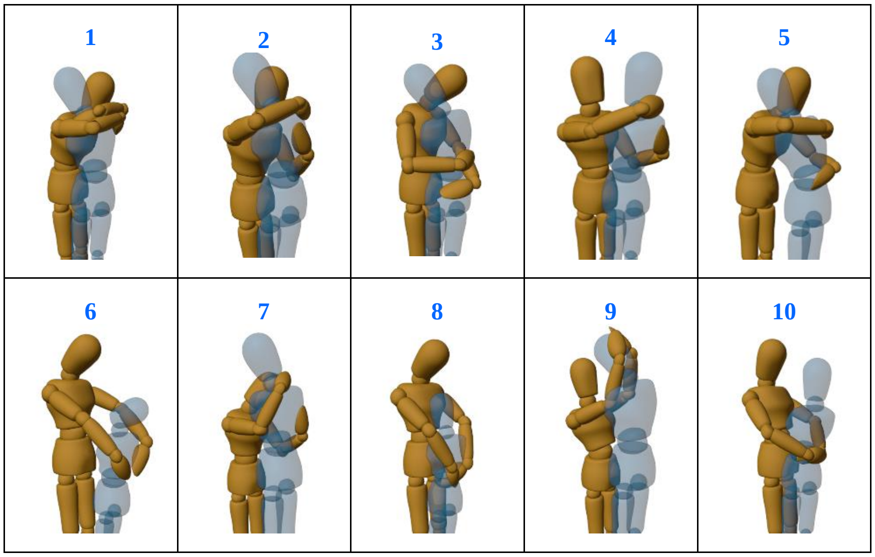

□如果是，选择此项即可（If so, select this option）

□如果否，请补充说明缺失的拥抱类型（If not, please describe the lack hug type）

____

Q5. 您认为这 10 个类别包含了您会完成的所有拥抱吗？（Do you believe these 10 categories encompass all the hugs you would provide?） [多选题] *

如下展示了拥抱类型的细节和描述（棕色木偶的姿势）
(The following figure shows the details and description of the hug types (the pose of the brown puppet).)

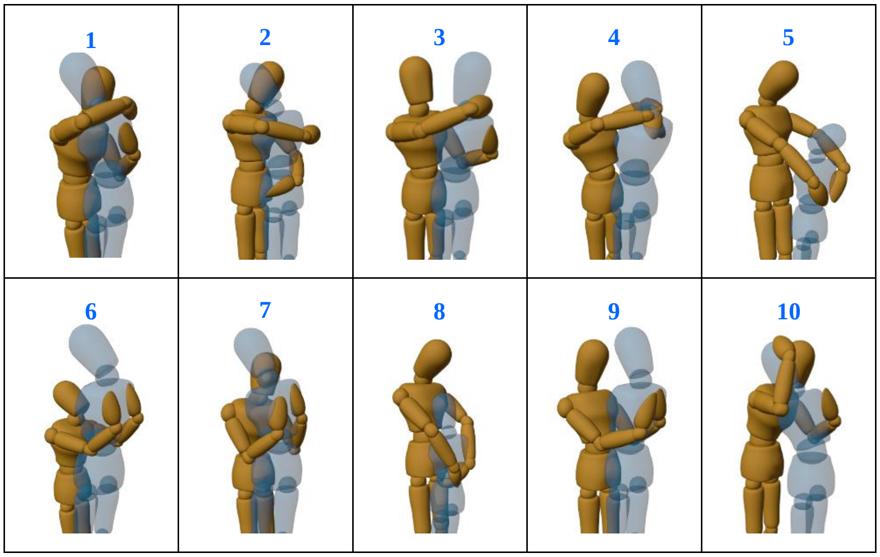

□如果是，选择此项即可（If so, select this option）

□如果否，请补充说明缺失的拥抱类型（If not, please describe the lack hug type）

____

Q6. 您认为这 12 个类别包含了您会完成的所有拥抱吗？（Do you believe these 12 categories encompass all the hugs you would provide?） [多选题] *

如下展示了拥抱类型的细节和描述（棕色木偶的姿势）
(The following figure shows the details and description of the hug types (the pose of the brown puppet).)

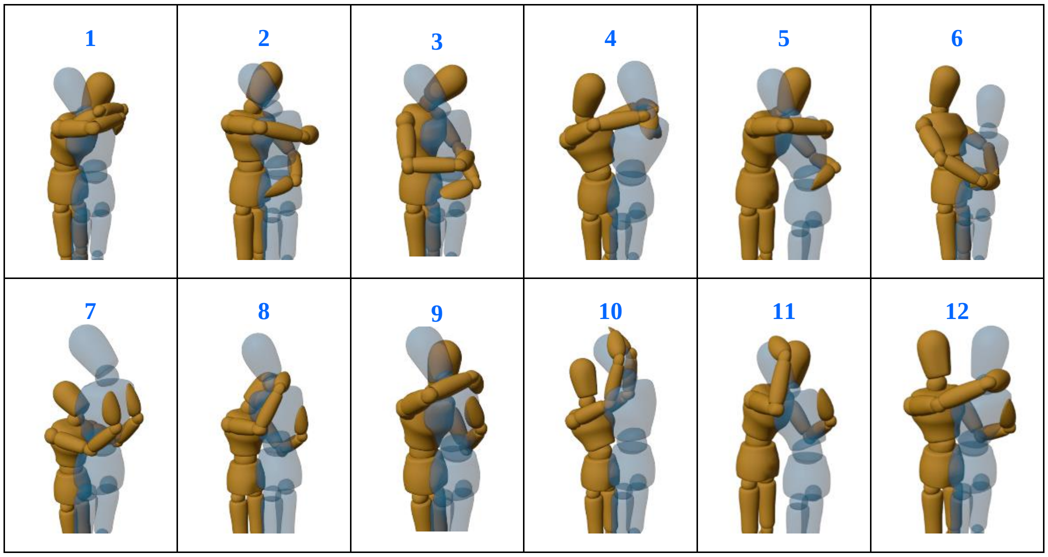

□如果是，选择此项即可（If so, select this option）

□如果否，请补充说明缺失的拥抱类型（If not, please describe the lack hug type）

Q7. 您认为这 10 个类别包含了您会完成的所有拥抱吗？（Do you believe these 10 categories encompass all the hugs you would provide?） [多选题] *

如下展示了拥抱类型的细节和描述（棕色木偶的姿势）

(The following figure shows the details and description of the hug types (the pose of the brown puppet).)

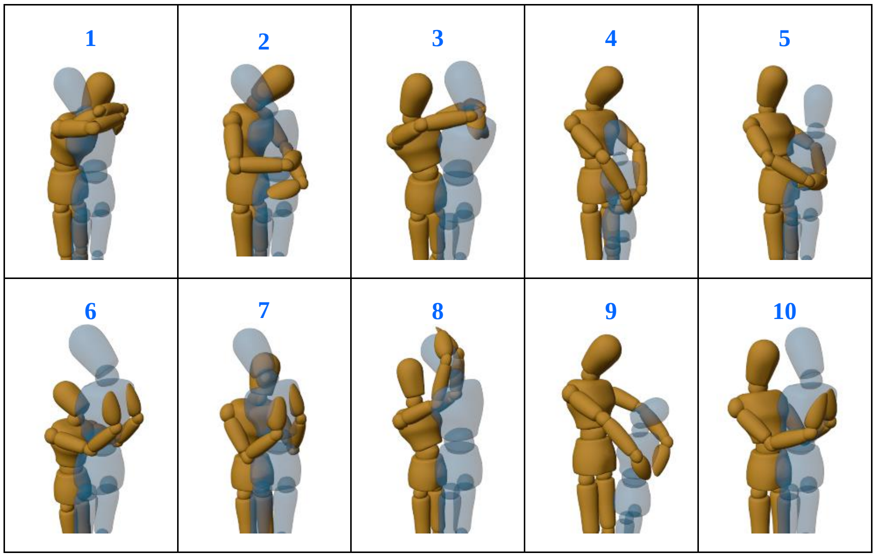

□如果是，选择此项即可（If so, select this option）

□如果否，请补充说明缺失的拥抱类型（If not, please describe the lack hug type）

____

Q8. 您认为这 10 个类别包含了您会完成的所有拥抱吗？（Do you believe these 10 categories encompass all the hugs you would provide?） [多选题] *

如下展示了拥抱类型的细节和描述（棕色木偶的姿势）
(The following figure shows the details and description of the hug types (the pose of the brown puppet).)

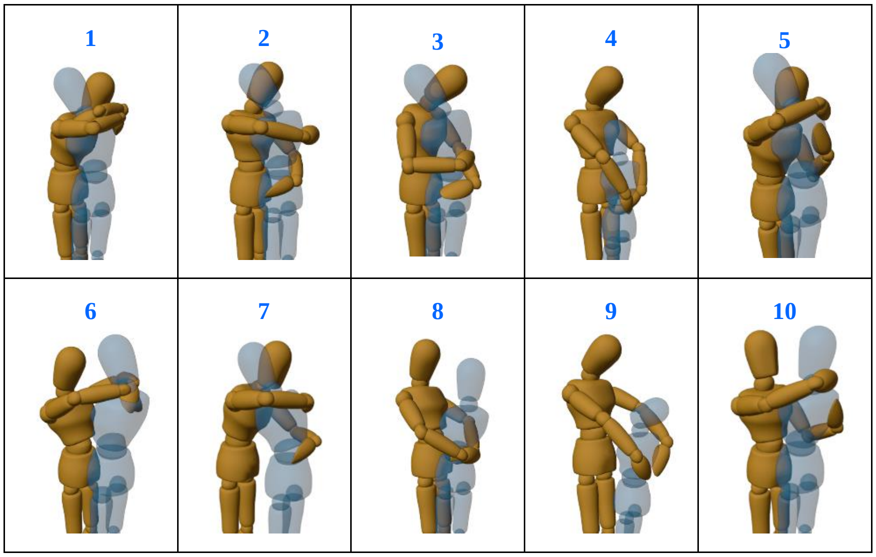

□如果是，选择此项即可（If so, select this option）

□如果否，请补充说明缺失的拥抱类型（If not, please describe the lack hug type）

____
