# Supplementary material for: A HUG taxonomy of humans with potential in human–robot hugs
Source: Sci Rep. 2024 Jun 20;14:14212. doi: 10.1038/s41598-024-64825-8 (PMC11190144; doi:10.1038/s41598-024-64825-8)
Supplement: Supplementary file 3 — Supplementary Information 3. [file 41598_2024_64825_MOESM3_ESM.pdf]

人类拥抱动作类型选择的调查

Survey of human hug type choice

1. 您的性别（Your gender） [单选题] *

○男（male）

○女（female）

2. 您的年龄（Your age） [填空题] *

____

3. 您是否对机器人感兴趣？（Are you interested in robots？） [单选题] *

○是（Yes）

○否（No）

4. 您是否有与机器人互动的经验？（Do you have experience about human-robot interaction?） [单选题] *

○是（Yes）

○否（No）

5. 您认为这 16 个类别包含了您会完成的所有拥抱吗？（Do you believe these 16 categories encompass all the hugs you would provide?） [多选题] *

如下展示了拥抱类型的细节和描述（棕色木偶的姿势）

(The following figure shows the details and description of the hug types (the pose of the brown puppet).)

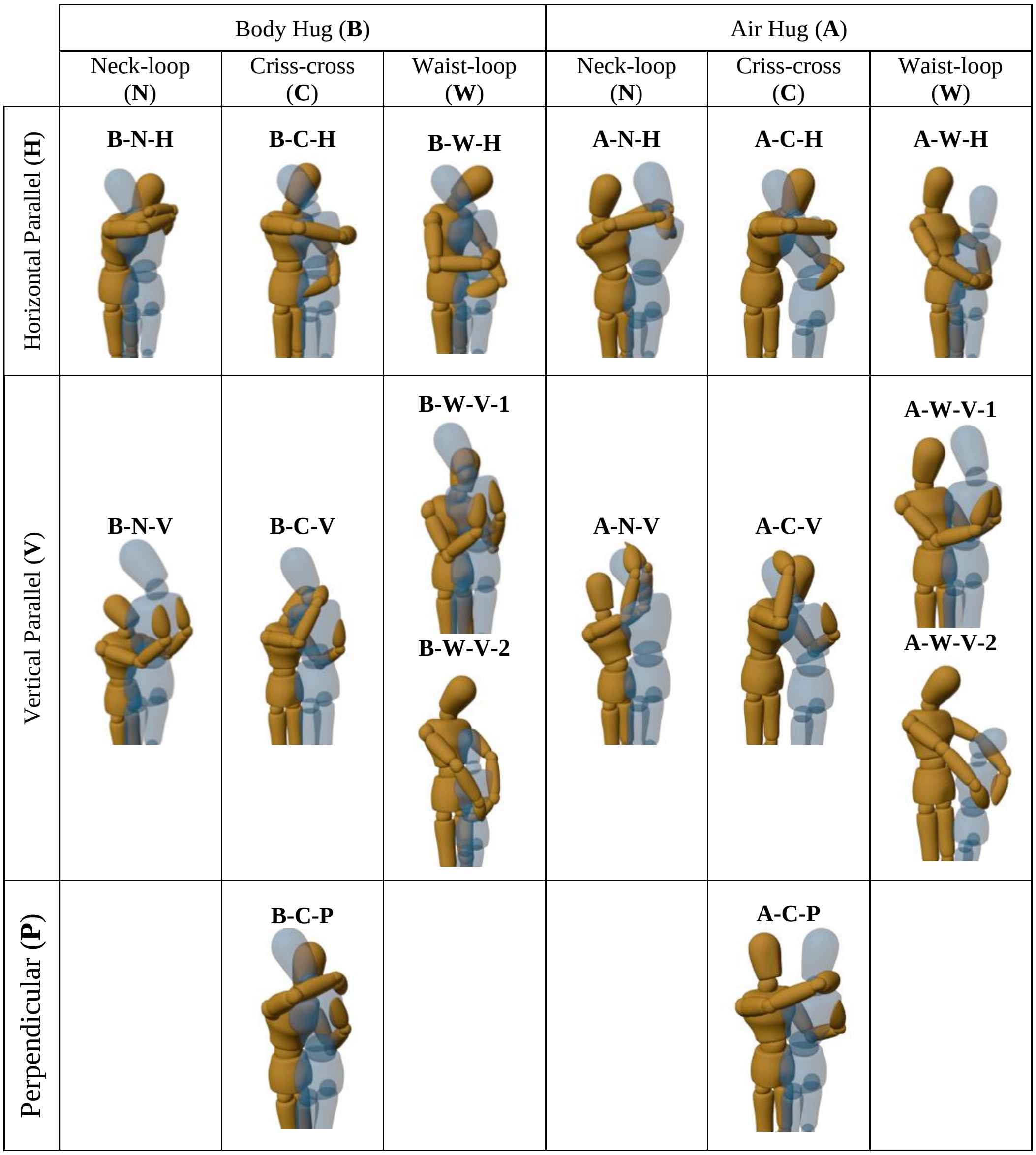

□如果是，选择此项即可（If so, select this option）

□如果否，请补充说明缺失的拥抱类型（If not, please describe the lack hug type）

____

6. 请您评估第 1 种拥抱类型，您是否会在如下情景中选择并主动发起？

Please evaluate the hug type 1, you tend to choose and initiate it in these scenarios.
[矩阵量表题] *

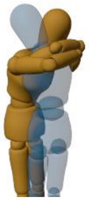

有胸部接触；大臂均向上；双臂水平成环

	非常不同意	不同意	中立	同意	非常同意
社交场合（会面、问候等）	○	○	○	○	○
亲密关系（情侣、家人等）	○	○	○	○	○
移动功能（旋转、平移等）	○	○	○	○	○
情绪表达（开心、悲伤等）	○	○	○	○	○

当有人使用该种拥抱类型拥抱您，您会优先选择哪些拥抱类型进行回应。请写下类型号。

When someone hugs you using this hug type, you prioritize to use which hug types for response. Please write down the hug type numbers.
[填空题] *

____

7. 请您评估第 2 种拥抱类型，您是否会在如下情景中选择并主动发起？

Please evaluate the hug type 2, you tend to choose and initiate it in these scenarios.
[矩阵量表题] *

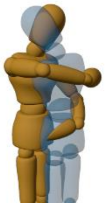
有胸部接触；大臂一上一下；双臂水平成环

	非常不同意	不同意	中立	同意	非常同意
社交场合（会面、问候等）	○	○	○	○	○
亲密关系（情侣、家人等）	○	○	○	○	○
移动功能（旋转、平移等）	○	○	○	○	○
情绪表达（开心、悲伤等）	○	○	○	○	○

当有人使用该种拥抱类型拥抱您，您会优先选择哪些拥抱类型进行回应。请写下类型号。

When someone hugs you using this hug type, you prioritize to use which hug types for response. Please write down the hug type numbers.
[填空题] *

____

8. 请您评估第 3 种拥抱类型，您是否会在如下情景中选择并主动发起？

Please evaluate the hug type 3, you tend to choose and initiate it in these scenarios.
[矩阵量表题] *

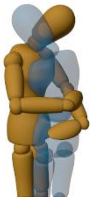

有胸部接触；大臂均向下；双臂水平成环

	非常不同意	不同意	中立	同意	非常同意
社交场合（会面、问候等）	○	○	○	○	○
亲密关系（情侣、家人等）	○	○	○	○	○
移动功能（旋转、平移等）	○	○	○	○	○
情绪表达（开心、悲伤等）	○	○	○	○	○

当有人使用该种拥抱类型拥抱您，您会优先选择哪些拥抱类型进行回应。请写下类型号。

When someone hugs you using this hug type, you prioritize to use which hug types for response. Please write down the hug type numbers.
[填空题] *

____

9. 请您评估第 4 种拥抱类型，您是否会在如下情景中选择并主动发起？

Please evaluate the hug type 4, you tend to choose and initiate it in these scenarios.
[矩阵量表题] *

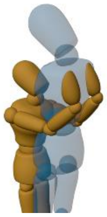

有胸部接触；大臂均向上；双臂竖直成环

	非常不同意	不同意	中立	同意	非常同意
社交场合（会面、问候等）	○	○	○	○	○
亲密关系（情侣、家人等）	○	○	○	○	○
移动功能（旋转、平移等）	○	○	○	○	○
情绪表达（开心、悲伤等）	○	○	○	○	○

当有人使用该种拥抱类型拥抱您，您会优先选择哪些拥抱类型进行回应。请写下类型号。

When someone hugs you using this hug type, you prioritize to use which hug types for response. Please write down the hug type numbers.
[填空题] *

____

10. 请您评估第 5 种拥抱类型，您是否会在如下情景中选择并主动发起？

Please evaluate the hug type 5, you tend to choose and initiate it in these scenarios.
[矩阵量表题] *

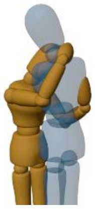

有胸部接触；大臂一上一下；双臂竖直成环

	非常不同意	不同意	中立	同意	非常同意
社交场合（会面、问候等）	○	○	○	○	○
亲密关系（情侣、家人等）	○	○	○	○	○
移动功能（旋转、平移等）	○	○	○	○	○
情绪表达（开心、悲伤等）	○	○	○	○	○

当有人使用该种拥抱类型拥抱您，您会优先选择哪些拥抱类型进行回应。请写下类型号。

When someone hugs you using this hug type, you prioritize to use which hug types for response. Please write down the hug type numbers.
[填空题] *

____

11. 请您评估第 6 种拥抱类型，您是否会在如下情景中选择并主动发起？

Please evaluate the hug type 6, you tend to choose and initiate it in these scenarios.
[矩阵量表题] *

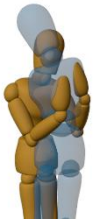
有胸部接触；大臂均向下；双臂竖直成环

	非常不同意	不同意	中立	同意	非常同意
社交场合（会面、问候等）	○	○	○	○	○
亲密关系（情侣、家人等）	○	○	○	○	○
移动功能（旋转、平移等）	○	○	○	○	○
情绪表达（开心、悲伤等）	○	○	○	○	○

当有人使用该种拥抱类型拥抱您，您会优先选择哪些拥抱类型进行回应。请写下类型号。

When someone hugs you using this hug type, you prioritize to use which hug types for response. Please write down the hug type numbers.
[填空题] *

____

12. 请您评估第 7 种拥抱类型，您是否会在如下情景中选择并主动发起？

Please evaluate the hug type 7, you tend to choose and initiate it in these scenarios.
[矩阵量表题] *

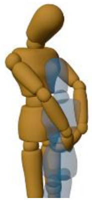
有胸部接触；大臂均向下；双臂竖直成环

	非常不同意	不同意	中立	同意	非常同意
社交场合（会面、问候等）	○	○	○	○	○
亲密关系（情侣、家人等）	○	○	○	○	○
移动功能（旋转、平移等）	○	○	○	○	○
情绪表达（开心、悲伤等）	○	○	○	○	○

当有人使用该种拥抱类型拥抱您，您会优先选择哪些拥抱类型进行回应。请写下类型号。

When someone hugs you using this hug type, you prioritize to use which hug types for response. Please write down the hug type numbers.
[填空题] *

____

13. 请您评估第 8 种拥抱类型，您是否会在如下情景中选择并主动发起？

Please evaluate the hug type 8, you tend to choose and initiate it in these scenarios.
[矩阵量表题] *

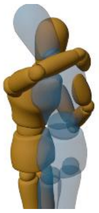

有胸部接触；大臂一上一下；双臂一水平环一竖直环

	非常不同意	不同意	中立	同意	非常同意
社交场合（会面、问候等）	○	○	○	○	○
亲密关系（情侣、家人等）	○	○	○	○	○
移动功能（旋转、平移等）	○	○	○	○	○
情绪表达（开心、悲伤等）	○	○	○	○	○

当有人使用该种拥抱类型拥抱您，您会优先选择哪些拥抱类型进行回应。请写下类型号。

When someone hugs you using this hug type, you prioritize to use which hug types for response. Please write down the hug type numbers.
[填空题] *

____

14. 请您评估第 9 种拥抱类型，您是否会在如下情景中选择并主动发起？

Please evaluate the hug type 9, you tend to choose and initiate it in these scenarios.
[矩阵量表题] *

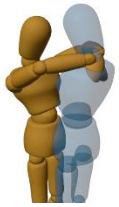

无胸部接触；大臂均向上；双臂水平成环

	非常不同意	不同意	中立	同意	非常同意
社交场合（会面、问候等）	○	○	○	○	○
亲密关系（情侣、家人等）	○	○	○	○	○
移动功能（旋转、平移等）	○	○	○	○	○
情绪表达（开心、悲伤等）	○	○	○	○	○

当有人使用该种拥抱类型拥抱您，您会优先选择哪些拥抱类型进行回应。请写下类型号。

When someone hugs you using this hug type, you prioritize to use which hug types for response. Please write down the hug type numbers.
[填空题] *

____

15. 请您评估第 10 种拥抱类型，您是否会在如下情景中选择并主动发起？

Please evaluate the hug type 10, you tend to choose and initiate it in these scenarios.
[矩阵量表题] *

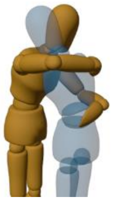
无胸部接触；大臂一上一下；双臂水平成环

	非常不同意	不同意	中立	同意	非常同意
社交场合（会面、问候等）	○	○	○	○	○
亲密关系（情侣、家人等）	○	○	○	○	○
移动功能（旋转、平移等）	○	○	○	○	○
情绪表达（开心、悲伤等）	○	○	○	○	○

当有人使用该种拥抱类型拥抱您，您会优先选择哪些拥抱类型进行回应。请写下类型号。

When someone hugs you using this hug type, you prioritize to use which hug types for response. Please write down the hug type numbers.
[填空题] *

____

16. 请您评估第 11 种拥抱类型，您是否会在如下情景中选择并主动发起？

Please evaluate the hug type 11, you tend to choose and initiate it in these scenarios.
[矩阵量表题] *

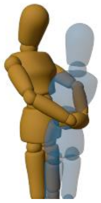
无胸部接触；大臂均向下；双臂水平成环

	非常不同意	不同意	中立	同意	非常同意
社交场合（会面、问候等）	○	○	○	○	○
亲密关系（情侣、家人等）	○	○	○	○	○
移动功能（旋转、平移等）	○	○	○	○	○
情绪表达（开心、悲伤等）	○	○	○	○	○

当有人使用该种拥抱类型拥抱您，您会优先选择哪些拥抱类型进行回应。请写下类型号。

When someone hugs you using this hug type, you prioritize to use which hug types for response. Please write down the hug type numbers.
[填空题] *

____

17. 请您评估第 12 种拥抱类型，您是否会在如下情景中选择并主动发起？

Please evaluate the hug type 12, you tend to choose and initiate it in these scenarios.
[矩阵量表题] *

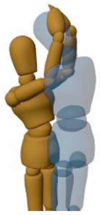

无胸部接触；大臂均向上；双臂竖直成环

	非常不同意	不同意	中立	同意	非常同意
社交场合（会面、问候等）	○	○	○	○	○
亲密关系（情侣、家人等）	○	○	○	○	○
移动功能（旋转、平移等）	○	○	○	○	○
情绪表达（开心、悲伤等）	○	○	○	○	○

当有人使用该种拥抱类型拥抱您，您会优先选择哪些拥抱类型进行回应。请写下类型号。

When someone hugs you using this hug type, you prioritize to use which hug types for response. Please write down the hug type numbers.
[填空题] *

____

18. 请您评估第 13 种拥抱类型，您是否会在如下情景中选择并主动发起？

Please evaluate the hug type 13, you tend to choose and initiate it in these scenarios.
[矩阵量表题] *

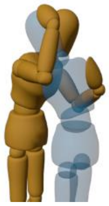

无胸部接触；大臂一上一下；双臂竖直成环

	非常不同意	不同意	中立	同意	非常同意
社交场合（会面、问候等）	○	○	○	○	○
亲密关系（情侣、家人等）	○	○	○	○	○
移动功能（旋转、平移等）	○	○	○	○	○
情绪表达（开心、悲伤等）	○	○	○	○	○

当有人使用该种拥抱类型拥抱您，您会优先选择哪些拥抱类型进行回应。请写下类型号。

When someone hugs you using this hug type, you prioritize to use which hug types for response. Please write down the hug type numbers.
[填空题] *

____

19. 请您评估第 14 种拥抱类型，您是否会在如下情景中选择并主动发起？

Please evaluate the hug type 14, you tend to choose and initiate it in these scenarios.
[矩阵量表题] *

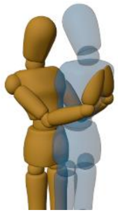

无胸部接触；大臂均向下；双臂竖直成环

	非常不同意	不同意	中立	同意	非常同意
社交场合（会面、问候等）	○	○	○	○	○
亲密关系（情侣、家人等）	○	○	○	○	○
移动功能（旋转、平移等）	○	○	○	○	○
情绪表达（开心、悲伤等）	○	○	○	○	○

当有人使用该种拥抱类型拥抱您，您会优先选择哪些拥抱类型进行回应。请写下类型号。

When someone hugs you using this hug type, you prioritize to use which hug types for response. Please write down the hug type numbers.
[填空题] *

____

20. 请您评估第 15 种拥抱类型，您是否会在如下情景中选择并主动发起？

Please evaluate the hug type 15, you tend to choose and initiate it in these scenarios.
[矩阵量表题] *

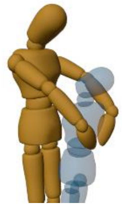

无胸部接触；大臂均向下；双臂竖直成环

	非常不同意	不同意	中立	同意	非常同意
社交场合（会面、问候等）	○	○	○	○	○
亲密关系（情侣、家人等）	○	○	○	○	○
移动功能（旋转、平移等）	○	○	○	○	○
情绪表达（开心、悲伤等）	○	○	○	○	○

当有人使用该种拥抱类型拥抱您，您会优先选择哪些拥抱类型进行回应。请写下类型号。

When someone hugs you using this hug type, you prioritize to use which hug types for response. Please write down the hug type numbers.
[填空题] *

____

21. 请您评估第 16 种拥抱类型，您是否会在如下情景中选择并主动发起？

Please evaluate the hug type 16, you tend to choose and initiate it in these scenarios.
[矩阵量表题] *

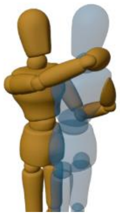
无胸部接触；大臂一上一下；双臂一水平环一竖直环

	非常不同意	不同意	中立	同意	非常同意
社交场合（会面、问候等）	○	○	○	○	○
亲密关系（情侣、家人等）	○	○	○	○	○
移动功能（旋转、平移等）	○	○	○	○	○
情绪表达（开心、悲伤等）	○	○	○	○	○

当有人使用该种拥抱类型拥抱您，您会优先选择哪些拥抱类型进行回应。请写下类型号。

When someone hugs you using this hug type, you prioritize to use which hug types for response. Please write down the hug type numbers.
[填空题] *

____
