# Supplementary material for: A HUG taxonomy of humans with potential in human–robot hugs
Source: Sci Rep. 2024 Jun 20;14:14212. doi: 10.1038/s41598-024-64825-8 (PMC11190144; doi:10.1038/s41598-024-64825-8)
Supplement: Supplementary file 5 — Supplementary Information 5. [file 41598_2024_64825_MOESM5_ESM.pdf]

The distribution of participants' involvement within all samples is as follows:

I / R	No. 1	No. 2	No. 3	No. 4	No. 5	No. 6	Total
No. 1		14	13	14	15	13	69
No. 2	15		14	14	13	13	69
No. 3	9	12		13	13	12	59
No. 4	8	13	12		11	12	56
No. 5	8	12	14	14		14	62
No. 6	10	13	15	15	11		64
Total	50	64	68	70	63	64	379

Where “**I**” represents the hug initiator and “**R**” represents the hug receiver.

When being the hug initiator, participant No.1 performed 50 hugs, participant No.2 performed 64 hugs, participant No.3 performed 68 hugs, participant No.4 performed 70 hugs, participant No.5 performed 63 hugs, and participant No.6 performed 64 hugs.

When being the hug receiver, participant No.1 performed 69 hugs, participant No.2 performed 69 hugs, participant No.3 performed 59 hugs, participant No.4 performed 56 hugs, participant No.5 performed 62 hugs, and participant No.6 performed 64 hugs.

In total, participant No.1 performed 119 hugs (15.7 %), participant No.2 performed 133 hugs (17.5 %), participant No.3 performed 127 hugs (16.8 %), participant No.4 performed 126 hugs (16.6 %), participant No.5 performed 125 hugs (16.5 %), and participant No.6 performed 128 hugs (16.9 %).

In the below five tables regarding each participant, the number of each hug type is represented as "m+n", where m represents the hug type counts initiated by the participant, and n represents the hug type counts received by the participant.

The distribution of hug types within all samples of participant No. 1 is as follows:

Hug type	A-C-H	A-C-P	A-N-H	A-N-V	A-W-H	A-W-V-1	A-W-V-2	A-C-V
Count	10+11	7+8	2+2	1+1	2+9	2+5	0+0	8+11
Percent	17.65%	12.61%	3.36%	1.68%	9.24%	5.88%	0.00%	15.97%
Hug type	B-C-H	B-C-P	B-N-H	B-N-V	B-W-H	B-W-V-1	B-W-V-2	B-C-V
Count	6+6	3+1	1+1	0+0	0+10	3+0	0+0	5+4
Percent	10.08%	3.36%	1.68%	0.00%	8.40%	2.52%	0.00%	7.56%

The distribution of hug types within all samples of participant No. 2 is as follows:

Hug type	A-C-H	A-C-P	A-N-H	A-N-V	A-W-H	A-W-V-1	A-W-V-2	A-C-V
Count	16+1	0+5	0+5	3+0	4+9	6+8	2+2	4+5
Percent	12.78%	3.76%	3.76%	2.26%	9.77%	10.53%	3.01%	6.77%
Hug type	B-C-H	B-C-P	B-N-H	B-N-V	B-W-H	B-W-V-1	B-W-V-2	B-C-V
Count	6+12	4+7	4+0	3+0	4+5	3+3	1+3	4+4
Percent	13.53%	8.27%	3.01%	2.26%	6.77%	4.51%	3.01%	6.02%

The distribution of hug types within all samples of participant No. 3 is as follows:

Hug type	A-C-H	A-C-P	A-N-H	A-N-V	A-W-H	A-W-V-1	A-W-V-2	A-C-V
Count	9+13	5+2	3+2	1+0	10+11	1+1	0+0	6+3
Percent	17.32%	5.51%	3.94%	0.79%	16.54%	1.57%	0.00%	7.09%
Hug type	B-C-H	B-C-P	B-N-H	B-N-V	B-W-H	B-W-V-1	B-W-V-2	B-C-V
Count	10+10	5+0	3+1	1+0	10+10	0+0	0+0	4+6
Percent	15.75%	3.94%	3.15%	0.79%	15.75%	0.00%	0.00%	7.87%

The distribution of hug types within all samples of participant No. 4 is as follows:

Hug type	A-C-H	A-C-P	A-N-H	A-N-V	A-W-H	A-W-V-1	A-W-V-2	A-C-V
Count	5+6	6+1	1+3	0+0	9+7	11+9	2+6	2+3
Percent	8.73%	5.56%	3.17%	0.00%	12.70%	15.87%	6.35%	3.97%
Hug type	B-C-H	B-C-P	B-N-H	B-N-V	B-W-H	B-W-V-1	B-W-V-2	B-C-V
Count	7+2	5+1	1+0	0+0	7+7	11+6	2+3	1+2
Percent	7.14%	4.76%	0.79%	0.00%	11.11%	13.49%	3.97%	2.38%

The distribution of hug types within all samples of participant No. 5 is as follows:

Hug type	A-C-H	A-C-P	A-N-H	A-N-V	A-W-H	A-W-V-1	A-W-V-2	A-C-V
Count	8+9	6+2	4+4	0+0	9+12	2+5	0+0	6+2
Percent	13.60%	6.40%	6.40%	0.00%	16.80%	5.60%	0.00%	6.40%
Hug type	B-C-H	B-C-P	B-N-H	B-N-V	B-W-H	B-W-V-1	B-W-V-2	B-C-V
Count	8+9	7+2	2+1	0+0	5+14	0+0	0+0	6+2
Percent	13.60%	7.20%	2.40%	0.00%	15.20%	0.00%	0.00%	6.40%

The distribution of hug types within all samples of participant No. 6 is as follows:

Hug type	A-C-H	A-C-P	A-N-H	A-N-V	A-W-H	A-W-V-1	A-W-V-2	A-C-V
Count	2+12	6+3	1+4	4+0	11+8	4+3	0+0	3+3
Percent	10.94%	7.03%	3.91%	3.13%	14.84%	5.47%	0.00%	4.69%
Hug type	B-C-H	B-C-P	B-N-H	B-N-V	B-W-H	B-W-V-1	B-W-V-2	B-C-V
Count	6+6	8+3	5+0	0+0	11+19	0+0	0+0	3+3
Percent	9.38%	8.59%	3.91%	0.00%	23.44%	0.00%	0.00%	4.69%

The distribution of hug types within all samples of all the participants is as follows:

Hug type	A-C-H	A-C-P	A-N-H	A-N-V	A-W-H	A-W-V-1	A-W-V-2	A-C-V
Count	102	51	31	10	101	57	12	56
Percent	13.46%	6.73%	4.09%	1.32%	13.32%	7.52%	1.58%	7.39%
Hug type	B-C-H	B-C-P	B-N-H	B-N-V	B-W-H	B-W-V-1	B-W-V-2	B-C-V
Count	88	46	19	4	102	26	9	44
Percent	11.61%	6.07%	2.51%	0.53%	13.46%	3.43%	1.19%	5.80%
