# Supplementary material for: A HUG taxonomy of humans with potential in human–robot hugs
Source: Sci Rep. 2024 Jun 20;14:14212. doi: 10.1038/s41598-024-64825-8 (PMC11190144; doi:10.1038/s41598-024-64825-8)
Supplement: Supplementary file 6 — Supplementary Information 6. [file 41598_2024_64825_MOESM6_ESM.pdf]

A total of six participants were asked to hug the humanoid robot in two situations:
1) The human serves as the hug initiator and the robot serves as the hug receiver.

Humans initiatively embrace the robot at any time as they wish. They are free to choose their type of hug. It is important to note that the robot will respond after the human approaches.

2) The human serves as the hug receiver and the robot serves as the hug initiator.

Since our robotic platform is inconvenient to move, the “approach” step in a hug is completed by the humans. However, the robot will actively perform a hug to invite the humans to approach. The type of hug for the robot is selected by the humans.

The preparations before each experiment include:

- Ensure that the activity area of the robot is large enough that no obstacles would interfere with the movement of the robot arm.
- Ensure that the robotic arm (UR3), all sensors, and actuators are functioning properly.
- Set the robot with the initiate posture.

Before all the experiments, the robot is standing with arms hanging beside the body as shown in Fig. 1.

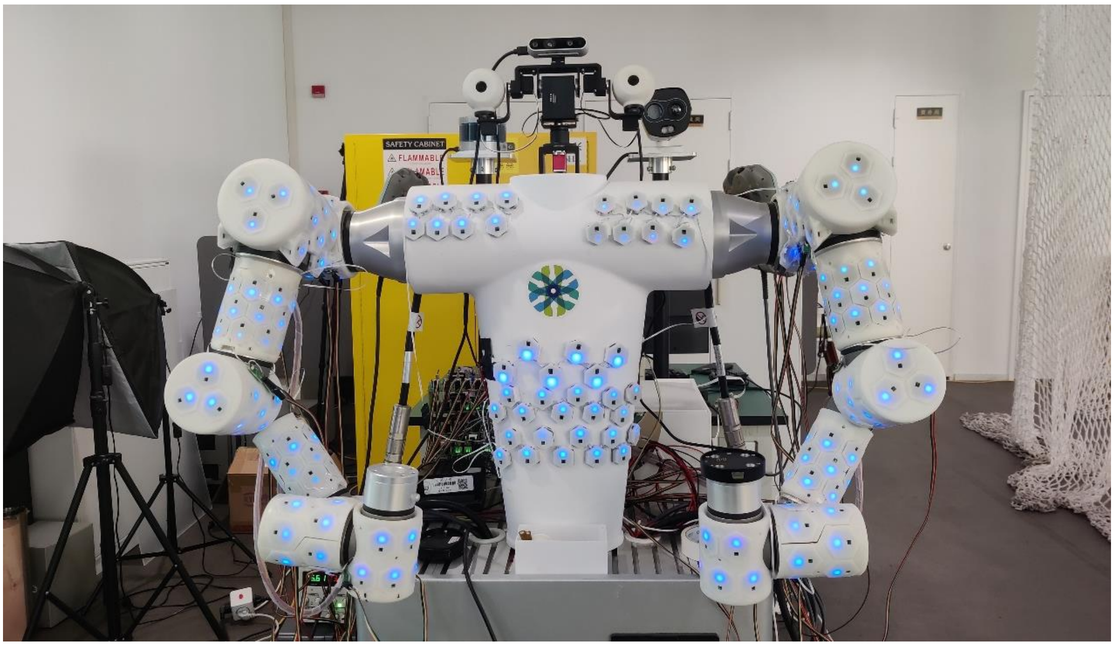

Fig. 1 The initial robot posture.

In each experiment, the complete process involves three steps: (1) robot's hug type selection, (2) kinesthetic teaching, and (3) practice. Next, we will introduce detailed information about each experiment step for two scenarios.

(1) Robot's hug type selection

The human operator of the robot determines the actions of the robot by interviewing participants. In each experiment, the human participant needs to first instruct the researcher on the type of hug, and then the researchers can continue the experimental process.

Human participants are asked to choose the robot's hug type based on their preferences.

For situation (1): They would be asked, “If you hug the robot, you will prefer which kind of hug type to be performed by the robot.”

For situation (2): They would be asked, “If the robot hugs you, you will prefer which kind of hug type to be performed by the robot.”

In this process, human participants may also be prompted by the four application scenarios (social occasion, intimate relationship, emotional expression, and movement functions).

The reason why we set up this guidance process is that human-robot interaction is a complex problem involving a wide range of factors. We believe that the focus of human-robot interaction is the human experience.

(2) Kinesthetic teaching

Embracing is usually considered to involve four stages, so our kinesthetic teaching implements the four stages in the following steps.

- The Approach

  For situation (1): The human participants approach the robot,

  For situation (2): The robot initiates a hug "invitation" for the approach of the participant.
- The Embrace, The Intra-hug Gestures, The release
  (1) The human operators hold the kinesthetic teaching button to make robotic arms perform the required motion.
  (2) The robotic control system records motion data, including the position of key points, and joint angles, in the hug process.
- The robotic arm control system replicates the movement trajectory based on the data during the recorded hug process.

Based on this process, the robot is enabled to hug the participant automatically.

(3) Practice

- After kinesthetic teaching, the participant practices with the robot for a hug, and then the human operator of the robot asks for the participant's evaluation.
- According to the evaluation, the human operator of the robot adjusts the robot's motion trajectory until the participant is satisfied with the experience.

During the practice, the robot should give a response at the right moment; the robot should not bump into the participant during the embrace; the hug should be comfortable for the human participants.

After the practice, we will record the data of the last hug which brings the best experience to the human participant.
